# Hedgehog signalling controls sinoatrial node development and atrioventricular cushion formation

**DOI:** 10.1098/rsob.210020

**Published:** 2021-06-02

**Authors:** Chaohui Zhang, Yuxin Li, Jiaheng Cao, Beibei Yu, Kaiyue Zhang, Ke Li, Xinhui Xu, Zhikun Guo, Yinming Liang, Xiao Yang, Zhongzhou Yang, Yunfu Sun, Vesa Kaartinen, Keyue Ding, Jikui Wang

**Affiliations:** ^1^ Henan Key Laboratory for Medical Tissue Regeneration, School of Basic Medical Sciences, Xinxiang Medical University, Xinxiang 453003, Henan Province, People's Republic of China; ^2^ Henan Collaborative Innovation Center of Molecular Diagnosis and Laboratory Medicine, School of Laboratory Medicine, Xinxiang Medical University, Xinxiang 453003, Henan Province, People's Republic of China; ^3^ State Key Laboratory of Proteomics, Beijing Institute of Lifeomics, Beijing 102206, People's Republic of China; ^4^ State Key Laboratory of Pharmaceutical Biotechnology, Model Animal Research Center, Nanjing University, Nanjing 210061, People's Republic of China; ^5^ Key Laboratory of Arrhythmia, Ministry of Education, East Hospital, Tongji University School of Medicine, Shanghai 200120, People's Republic of China; ^6^ Department of Biologic and Materials Sciences, School of Dentistry, University of Michigan, Ann Arbor, MI 48109, USA; ^7^ Medical Genetic Institute of Henan Province, Henan Provincial People's Hospital, People's Hospital of Zhengzhou University, Henan Provincial People's Hospital of Henan University, Zhengzhou 450003, People's Republic of China

**Keywords:** inflow tract, sinoatrial node, atrioventricular cushion, hedgehog signalling, *Smoothened*, *Tbx5*

## Abstract

Smoothened is a key receptor of the hedgehog pathway, but the roles of Smoothened in cardiac development remain incompletely understood. In this study, we found that the conditional knockout of *Smoothened* from the mesoderm impaired the development of the venous pole of the heart and resulted in hypoplasia of the atrium/inflow tract (IFT) and a low heart rate. The blockage of *Smoothened* led to reduced expression of genes critical for sinoatrial node (SAN) development in the IFT. In a cardiac cell culture model, we identified a Gli2–Tbx5–Hcn4 pathway that controls SAN development. In the mutant embryos, the endocardial-to-mesenchymal transition (EndMT) in the atrioventricular cushion failed, and Bmp signalling was downregulated. The addition of Bmp2 rescued the EndMT in mutant explant cultures. Furthermore, we analysed *Gli2^+^* scRNAseq and *Tbx5^−/−^* RNAseq data and explored the potential genes downstream of hedgehog signalling in posterior second heart field derivatives. In conclusion, our study reveals that Smoothened-mediated hedgehog signalling controls posterior cardiac progenitor commitment, which suggests that the mutation of *Smoothened* might be involved in the aetiology of congenital heart diseases related to the cardiac conduction system and heart valves.

## Graphical abstract

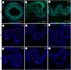

## 1. Introduction

The heart, as the first functional organ during development, serves as a pump that delivers nutrients and oxygen to the embryo. Cardiac progenitor formation and differentiation are essential for heart development. During early gastrulation, a subset of mesodermal cells leaves the primitive streak and is destined to a cardiac fate [1]. Later during development, at approximately E7.5, the lateral anterior splanchnic mesoderm forms crescent-shaped clusters of cells consisting of the first and second heart fields (first heart field: FHF; second heart field: SHF) [2,3]. The SHF lies medial and dorsal to the FHF. As cardiac development proceeds, the bilateral progenitors coalesce at the ventral midline and form a primitive heart tube. The heart tube elongates and loops through the addition of SHF progenitors from the arterial and venous poles [3,4]. The progenitors in the anterior SHF (aSHF) give rise to the right ventricle and outflow tract (OFT) at the arterial pole, whereas the posterior SHF (pSHF) progenitors contribute to the posterior portion of the heart, which includes the atrioventricular (AV) canal, atria and inflow tract (IFT) at the venous pole [5–7].

The hedgehog (Hh) pathway has been implicated in cardiac development in mammals through activation of Smoothened (SMO)-mediated downstream signalling events. *Smo^−/−^* mutant embryos fail to turn and are arrested at approximately E9.0 with a linear heart tube [8]. A global removal of *Shh* or the inactivation of *Smo* with *Mef2c^Cre/+^* in aSHF or with *Gli1^CreERT2^* leads to atrial septal defects due to loss of the dorsal mesenchymal protrusion (DMP) [9,10]. It has been reported that Tbx5 acts upstream or parallel to Hh signalling in cardiac progenitors and controls DMP formation at E10.5 [11]. Lineage tracing has indicated that Hh-receiving cells labelled at E6.5–E7.5 contribute to the AV canal, common atrium and IFT and to the other cardiac portions [12]. DiI labelling and clonal analysis has revealed that cardiac progenitors in pSHF contribute to the AV canal, atrium and IFT [5]. However, the function of Hh signalling in the pSHF during the development of the posterior portion of the heart remains incompletely elucidated.

In this study, we determined the role of Hh signalling in the cardiac mesoderm during early cardiac development. We used *Mesp1^Cre/+^* to abrogate the activity of *Smo* in the murine cardiac mesoderm. The inactivation of *Smo* resulted in hypoplasia of the IFT, common atrium and AV cushion. The mutant embryos also exhibited a low heart rate. We found that the loss of *Smo* impaired the developmental potential of cardiac progenitors due to downregulation of *Tbx5* in the pSHF. Genes critical for sinoatrial node (SAN) development were downregulated in the IFT of the mutant hearts. A Gli2–Tbx5–Hcn4 axis required for SAN development was identified. We also found that *Bmp2* expression was decreased in the mutant AV canal myocardium, and in explant cultures, the endocardial-to-mesenchymal transition (EndMT) defect was rescued by treatment with Bmp2. Moreover, we analysed *Gli2^+^* scRNAseq and *Tbx5^−/−^* RNAseq data and explored the potential genes downstream of Gli2 that are associated with cardiac contraction.

## 2. Material and methods

### 2.1. Animals

*Smo^flox/+^* (*Smo^F/+^*) (JAX: 004526), *Mesp1^Cre/+^* (Cat#: RBRC01145) and *Tie2^Cre/+^* animals were previously generated and maintained on a 129, TT2/ICR and B6/KM genetic background, respectively [1,13,14]. To specifically inactivate *Smo* in the mesoderm, we bred *Smo^F/+^*;*Mesp1^Cre/+^* animals with *Smo^flox/flox^* (*Smo^F/F^*) animals to generate *Smo^F/F^*;*Mesp1^Cre/+^* mutant embryos. To abrogate *Smo* in the endothelium, we bred *Smo^F/+^*;*Tie2^Cre/+^* animals with *Smo^F/F^* animals to generate *Smo^F/F^*;*Tie2^Cre/+^* mutant embryos. In all related experiments, control refers to stage-matched embryos that are either *Cre*(*+*) and *F/+*, or *F/F* but *Cre*(−), unless otherwise specified. Noon on the day at which a vaginal plug was observed was regarded as embryonic day 0.5 (E0.5). The embryonic stages for each experiment are indicated in the figures or legends, and the embryo sexes were unknown at the time of harvest. All the animals were housed in a pathogen-free environment, and all the animal experiments were performed according to a protocol approved by the Institutional Animal Care and Use Committee of Xinxiang Medical University.

### 2.2. Dissection, histology and immunostaining

Embryos at desired stages were dissected in either cold diethyl polycarbonate (DEPC)-treated phosphate-buffered saline (PBS) or room-temperature PBS and fixed for 2–16 h in 4% paraformaldehyde (PFA) at 4°C. The embryos were then dehydrated through an ethanol gradient, cleared with xylene, oriented and embedded in paraffin. Subsequently, the embryos were cut into serial sections and stained with hematoxylin and eosin (H&E). Immunostaining was performed according to the manufacturer's instructions. The sections were subjected to antigen retrieval before the application of blocking reagents and subsequent primary antibodies. Primary antibody information is provided in electronic supplementary material, table S1.

### 2.3. EdU assay

Timed pregnant mice received an IP injection of EdU (Ribobio) 2 h prior to embryo dissection. Immunostaining of EdU was performed on paraffin serial sections according to the manufacturer's instructions. EdU kit information is provided in the electronic supplementary material, table S1.

### 2.4. Whole-mount *in situ* hybridization

Whole-mount and section *in situ* hybridization (ISH) were performed as previously described [15,16]. Mouse DNA templates (*Tbx5*, *Wnt2*, *Hcn4*, *Isl1*, *Nkx2.5*, *Myl7*, *Meis1*, *Arid3b*, *Bmp2* and *Twist1*) were amplified by PCR from corresponding cDNA and subcloned into the pBlueScriptSK or pCR2.1 vector with the indicated primers and used to generate probes (electronic supplementary material, table S1); the plasmids are available upon request. After fixation, the embryos or sections were treated with 10 µg ml^−1^ proteinase K, re-fixed in 4% PFA/0.2% glutaraldehyde solution and prehybridized twice at 68°C for 30 min. The specimens were then hybridized overnight at 70°C with digoxigenin (DIG)-labelled antisense RNA probes. The following day, the embryos/sections were washed, blocked and incubated overnight with alkaline phosphatase (AP)-conjugated anti-DIG IgG. AP activity was detected using BM purple (Roche). The embryos/sections were postfixed in 4% PFA/0.2% glutaraldehyde prior to visualization.

### 2.5. Quantitative RT-PCR (qRT-PCR)

Total RNA was isolated from IFT and cultured cells with TriPure (Roche) and converted to cDNA with a SuperScript III cDNA Synthesis Kit (Invitrogen) according to the manufacturer's instructions. The primers were selected from PrimerBank or self-designed (electronic supplementary material, table S1). qPCR was performed using SYBR Green, and the relative expression level was normalized to β-actin using the ΔΔCt method.

### 2.6. Explant culture

Explant culture was performed according to a previous report [17]. AV canals from E9.5 hearts were dissected and cultured on collagen gels for up to 50 h. For rescue assays, Bmp2 (100 ng ml^−1^) was added to the culture medium. The dissection and explant culture were repeated at least three times.

### 2.7. Measurement of heart rate

E9.5 heart tubes (or embryos) were dissected in DMEM containing 10% FBS, penicillin (100 units ml^−1^) and streptomycin (100 units ml^−1^). The heart tubes or hearts were then transferred to a prewarmed medium (37°C) and incubated in a humidified incubator (supplied with 5% CO_2_ and 95% air) for 1 h. After incubation, the beating heart tubes (or hearts) were taken out for video recording, and the heart rates were measured.

### 2.8. Cell culture

P19CL6 cells were cultured as previously described and differentiated in 1% dimethyl sulfoxide (DMSO) [18]. Briefly, the cells were maintained in an α-minimal essential medium (Thermo Fisher) supplemented with 10% fetal calf serum (HyClone), 4 mM l-glutamine, penicillin (100 units ml^−1^) and streptomycin (100 units ml^−1^) at 37°C in a humidified incubator containing 5% CO_2_ and 95% air. To induce cardiac differentiation, 1% DMSO was added to the P19CL6 culture medium. For transient transfection, P19CL6 cells were cultured in a differentiation medium for 2 days. On the third day, gene overexpression assays were conducted via the transfection of *Gli2* (p*CEFL3xHAmGli2* [19]) *Gli1* (*pcCDNA3.1-Gli1*, YouBio) or *Tbx5* (*pTbx5-IRES-hrGFPII*, homemade, the mouse *Tbx5* coding sequence was cloned into IRES-hrGFPII) using Lipofectamine 3000 (Thermo Fisher) according to the instruction manual. Briefly, after 2 days of differentiation, P19CL6 cells were seeded into a 24-well culture plate at a density of 1.5 × 10^5^ well^−1^. After 12–14 h, the cells were transfected with 500 ng of p*CEFL3xHAmGli2*, *pcDNA3.1-Gli1* or *pTbx5-IRES-hrGFPII* and incubated for another 48 h under differentiation conditions before harvest. Cells transfected with the empty vector or vehicle were used as control. For the Smoothened inhibition assay, the transfection step was replaced by the addition of sonidegib (working concentration: 10 µM) to the cells in a 24-well plate. The cells were tested, and no mycoplasma contamination was found. All cell assays were performed in duplicate or triplicate, and the experiments were repeated at least three times.

### 2.9. Bioinformatic analysis

Gene ontology (GO) expression analysis was performed using the DAVID Bioinformatics Resources and WEB-based GEne SeT AnaLysis Toolkit.

### 2.10. Statistical analysis

All the data are presented as the means ± SEMs from at least three independent experiments. Unpaired two-tailed Student's *t*-tests or Mann–Whitney tests were used for the statistical analyses.

## 3. Results

### 3.1. Smo and its main downstream transcription factors are expressed in the cardiac mesoderm

**Figure 1.**
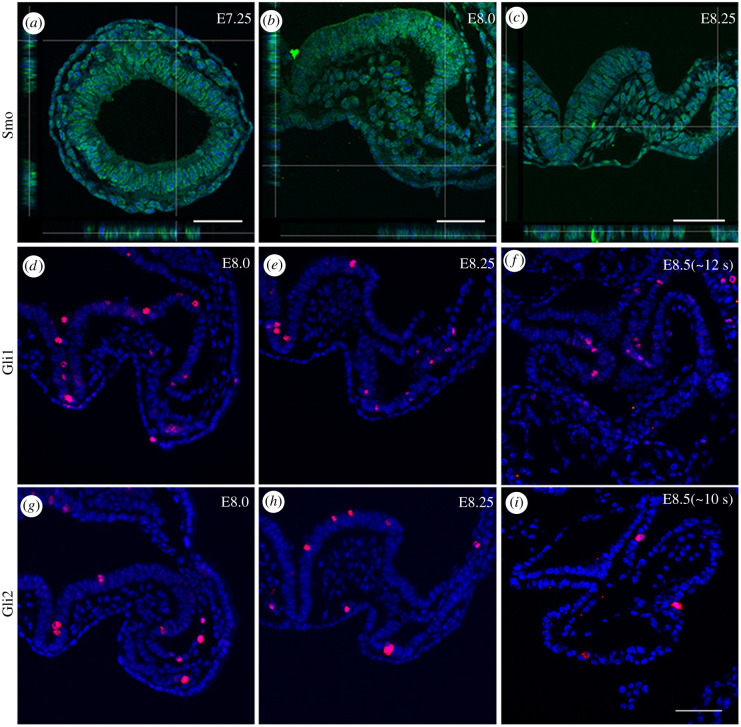
Expression of the main components of the Hh pathway during normal development. (*a*–*c*) Smo was expressed in the nascent mesodermal germ layer and the cardiac mesoderm. Smo: green; DAPI: blue. (*d*–*f*) Gli1 was expressed in the cardiac mesoderm, atrium/IFT and dorsal mesoderm. Gli1: red; DAPI: blue. (*g*–*i*) Gli2 was expressed in the cardiac mesoderm, atrium and dorsal mesoderm. Gli2: red; DAPI: blue. Scale bars: 50 µm.

We first analysed the expression patterns of Smo and its downstream transcriptional factors in the early developing mouse. At E7.0–E7.5, Smo and its downstream transducers Gli1 and Gli2 were observed in the mesodermal germ layer (Smo: figure 1*a*; Gli1 and Gli2: electronic supplementary material, figure S1A–C) and in other germ layers. By E8.0–E8.25, Smo, Gli1 and Gli2 were expressed in the cardiac mesoderm (figure 1*b*,*c*,*d*,*e*, and *g*,*h*, respectively). Although the expression of Smo was barely detected by E8.5 (electronic supplementary material, figure S1D–D″), Gli1 and Gli2 expression was present in the atrium/IFT and the connected dorsal mesoderm at E8.5 (figure 1*f*,*i*).

### 3.2. Hh signalling is required for IFT and common atrium development and AV cushion formation

To determine the function of Hh signalling in cardiac progenitors, we specifically abrogated Smo activity in the mesoderm using *Mesp1^Cre^*^/+^ mice.

**Figure 2.**
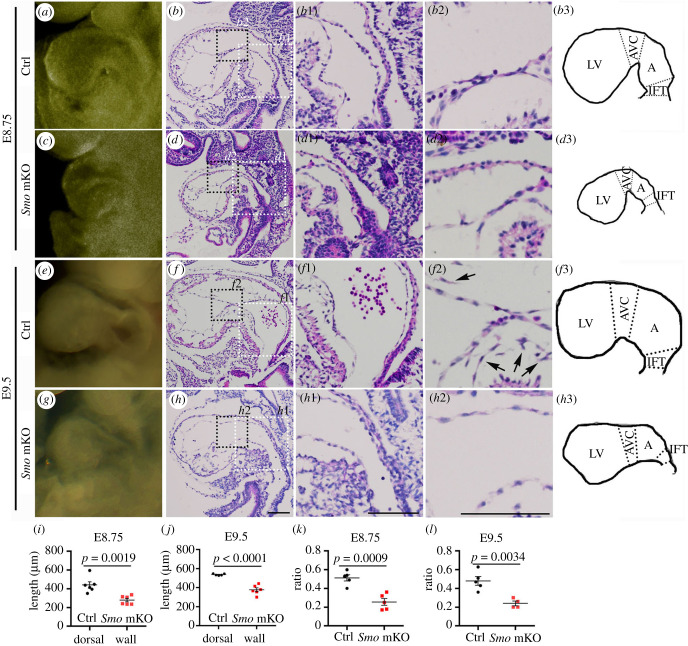
Hypoplastic defects in the IFT, atrium and AV cushion of the *Smo* mKO mutant embryos. (*a*–*d*3) Gross morphology and H&E staining of midsagittal sections of the control and *Smo* mKO mutant hearts at E8.75 showing the IFT (Ctrl: *a*,*b*,*b*1; Mut: *c*,*d*,*d*1), atrium (Ctrl: *a*,*b*,*b*1; Mut: *c*,*d*,*d*1) and AV cushion (Ctrl: *b*,*b*2; Mut: *d*,*d*2). *b*3 and *d*3 show cartoon illustrations of the cardiac anatomical structure in the control and mutant hearts at E8.75, respectively. (*e*–*h*3) Gross morphology and H&E staining of midsagittal sections of the control and *Smo* mKO mutant hearts at E9.5 showing the IFT (Ctrl: *e*,*f*,*f*1; Mut: *g*,*h*,*h*1), atrium (Ctrl: *e*,*f*,*f*1; Mut: *g*,*h*,*h*1) and AV cushion (Ctrl: *f*,*f*2; Mut: *h*,*h*2). *f*3 and *h*3 show cartoon illustrations of the cardiac anatomical structure in the control and mutant hearts at E9.5, respectively. The arrows in *f*2 indicate mesenchymal cells in the AV cushion. (*i*–*j*) Statistical comparison of the length of the dorsal myocardial wall from the base of the IFT to the junction between the ventricle and AV canal (*i*: E8.75; *j*: E9.5). (*k*–*l*) Sizes of the IFT and atrium relative to that of the right ventricle in the control and *Smo* mKO mutant embryos (*k*: E8.75; *l*: E9.5). Scale bars: 100 µm. The histogram shows the means ± SEMs.

*Smo^F/F^*;*Mesp1^Cre/+^* (*Smo* mKO) mutant embryos were grossly indistinguishable from their littermate controls by E8.5. At E8.75, although embryonic turning and gut tube closure appeared to be normal (electronic supplementary material, figure S2), a small primitive atrium was observed in the mutant heart (figure 2*a*,*c*). The gross atrial defect was present after E9.5 (figure 2*e*,*g*). Moreover, the OFT and right ventricle derived from the anterior secondary heart field and the left ventricle derived from FHF exhibited a reduction in size and impaired cardiac looping as the embryo developed (electronic supplementary material, figure S3). The survival rate indicated that the viability of the mutant embryos began to decline at E10.5 (electronic supplementary material, table S2).

We then conducted a histological analysis of the hearts at different stages. In this study, we were particularly interested in pSHF development. Serial sections indicated that the mutant embryos had smaller AV canals and atria and shorter IFTs than the controls at E8.75 (approx. 16 somite stage) and E9.5 (figure 2*b*,*d*,*b*1,*d*1 and *f*,*h*,*f*1,*h*1, respectively). At E8.75, mesenchymal cells were barely detectable in the AV cushions of both the control and mutant embryos (figure 2*b*2,*d*2). By E9.5, mesenchymal cells had formed in the AV cushions of the control embryos (figure 2*f*,*f*2). However, no or very few mesenchymal cells were found in the mutant AV cushions (figure 2*h*,*h*2).

We further quantitatively assessed the morphological defects of the posterior portions of the developing hearts. The lengths of the dorsal myocardial walls (IFT + atrium + AV canal, midsagittal section) were significantly decreased in the mutant hearts (E8.75, control: 404.70 ± 34.07 µm, mutant: 278.7 ± 18.77 µm, *n* = 6, *p* = 0.0019; E9.5, control: 537.00 ± 4.11 µm, mutant: 376.00 ± 20.35 µm, *n* = 5–6, *p* < 0.0001) (figure 2*i*,*j*). We also measured the areas of the ventricle, atrium and IFT in midsagittal sections. The area ratios for the atrium and IFT relative to the left ventricle were significantly smaller in the mutant hearts than in the controls (E8.75, control: 0.5120 ± 0.0331, mutant: 0.2560 ± 0.0375, *n* = 5, *p* = 0.0009; E9.5, control: 0.4800 ± 0.0450, mutant: 0.2400 ± 0.0250, *n* = 4–5, *p* = 0.0034) (figure 2*k*,*l*).

Taken together, these results demonstrate that mesodermal *Smo* controls atrial and IFT development and AV cushion formation in developing hearts.

### 3.3. Loss of *Smo* in the mesoderm impairs the developmental potentials of cardiac progenitors in the pSHF

**Figure 3.**
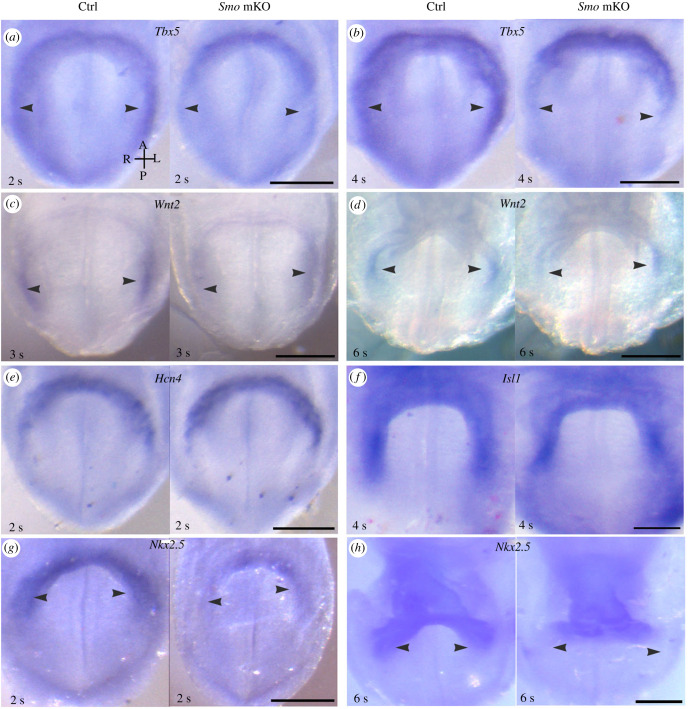
Impaired developmental potentials of cardiac progenitors in the pSHF of *Smo* mKO mutants. (*a*,*b*) Downregulated expression of *Tbx5* in the posterior cardiac field of the *Smo* mKO mutants (arrowheads). (*c*,*d*) Downregulated expression of *Wnt2* in the pSHF (arrowheads) of the mutants. (*e*,*f*) Relatively normal expression of *Hcn4* and *Isl1* in the FHF and SHF, respectively, of the mutant embryos. (*g*,*h*) Downregulated expression of *Nkx2.5* in the posterior cardiac field of the *Smo* mKO mutants (arrowheads). Scale bars: 100 µm.

Given that the *Smo* mKO mutants phenocopy *Tbx5* homozygous mutants with respect to the posterior developing heart (i.e. the primitive atrium and IFT) [20], we examined the expression of *Tbx5* in early mutant embryos. Whole-mount ISH showed that *Tbx5* expression was reduced in the posterior portion of the cardiac crescent at the 2–4 s stage (approx. E8.0) (figure 3*a*,*b*). *Wnt2* is regulated by Tbx5 and is required for development of the cardiac posterior pole [21]. At E8.0–E8.25, the expression of *Wnt2* was reduced in the *Smo* mKO mutants (figure 3*c*,*d*).

*Hcn4* (hyperpolarization-activated cyclic nucleotide-gated potassium channel 4) is a marker of the FHF and expressed at the cardiac crescent at the approximately 2–4s stage [22]. *Tbx5* and *Hcn4* expression domains mostly overlap in the FHF [23]. Whole-mount ISH indicated that *Hcn4* expression in the cardiac crescent of the controls was comparable to that found in the *Smo* mKO mutants (figure 3*e*) at the approximately 2 s stage.

*Isl1* marks the SHF during cardiogenesis [24]. At the approximately 4 s stage, *Isl1* and *Tbx5* show overlap in their posterior expression domains [23]. The expression of *Isl1* in the *Smo* mKO mutants did not differ from that in the controls (figure 3*f*).

*Nkx2.5* marks cardiac progenitors in both the FHF and the SHF, and its expression is maintained beyond birth. In the *Smo* mKO mutants, the expression of *Nkx2.5* was downregulated at E8.0 in the cardiac crescent and later in the sinus venosus (figure 3*g*,*h*), and by E9.5, *Nkx2.5* expression returned to a normal level (electronic supplementary material, figure S4). These results are consistent with those found in the *Smo*^−/−^ mutants [8]. We then examined the expression of MF20, a myosin heavy chain protein, by immunostaining and found no difference between the controls and mutants (electronic supplementary material, figure S5).

Thus, Smo is required for the expression of *Tbx5* and *Nkx2.5*, but not *Isl1* and *Hcn4*, in the cardiac progenitors located in the posterior cardiac crescent. The results demonstrate that Hh signalling controls the developmental potentials, not the formation, of the cardiac progenitors in the pSHF.

### 3.4. Loss of Smo activation in the pSHF impairs the development and function of the SAN

**Figure 4.**
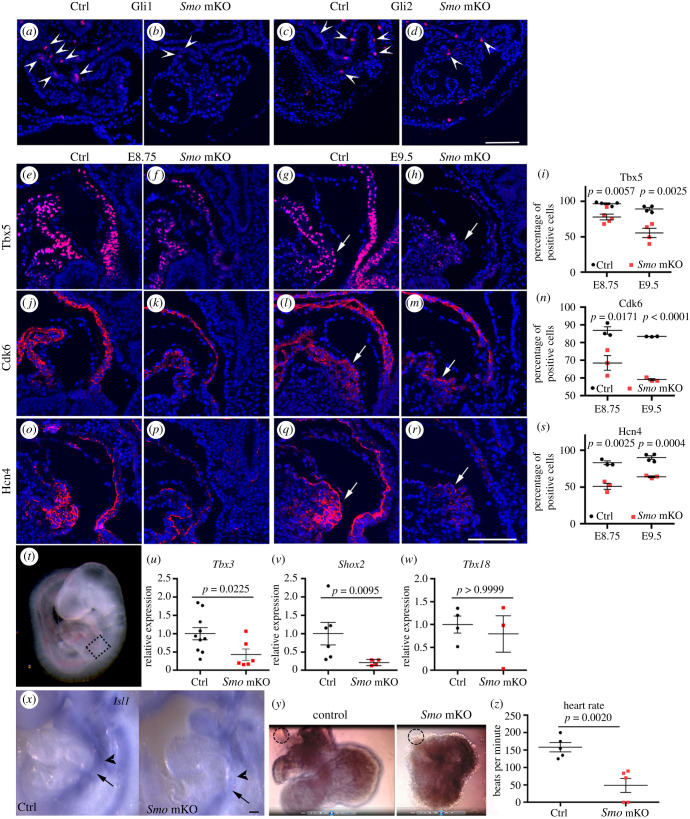
Impaired development of the cardiac conduction system in the IFT and atrium of the *Smo* mKO mutants. (*a*,*b*) Reduced expression of Gli1 in the atrium/IFT and dorsal mesoderm (pSHF) of the *Smo* mKO mutants at E8.75 (arrowheads). (*c*,*d*) Reduced expression of Gli2 in the atrium/IFT and pSHF of the *Smo* mKO mutants at E8.75 (arrowheads). (*e*,*i*) Reduced expression of Tbx5 in the IFT and atrium of the mutants (arrows: putative SAN primordium). (*j*–*n*) Reduced expression of Cdk6 in the IFT and atrium of the mutants (arrows: putative SAN primordium). (*o*–*s*) Reduced expression of Hcn4 in the IFT and atrium of the mutants (arrows: putative SAN primordium). (*t*) Embryo showing the cardiac IFT dissected for qRTPCR. The dashed box indicates the IFT of E9.0 embryos. (*u*–*w*) qRT-PCR analysis of *Tbx3*, *Shox2* and *Tbx18* in the IFT of the *Smo* mKO embryos. (*x*) Downregulated expression of *Isl1* in the IFT and pSHF of the mutant embryos (arrow: IFT, arrowhead: pSHF). (*y*,*z*) Reduced heartbeats in the mutant embryos (*y*: control and mutant hearts in culture from videos; *z*: statistics of the heart rate, dashed circle: putative sinoatrial node). Scale bar: 100 µm.

We assessed the activities of Hh signalling in the *Smo* mKO mutants. Gli1 is a transcription activator and amplifies the exiting Hh signalling, and it has been reported that *Gli1* is a direct transcriptional target of Gli2 [25]. Mouse genetic studies have shown that Gli2 mainly functions as a strong activator in response to Hh signalling [26]. In *Smo* mKO mice, the expression of Gli1 and Gli2 in the atrium/IFT was downregulated (figure 4*a–d*). These results suggested that Hh signalling was repressed in the *Sm*o mKO mutant hearts.

The IFT and atrium are mainly derived from the pSHF. *Tbx5* expression patterns the IFT and atrium at the cardiac looping stage [27]. Hypomorphic *Tbx*5*^lox/+^* mice display sinus rhythm with premature atrial complexes and sinoatrial pauses [28]. In the *Smo* mKO mutant mice, the expression of Tbx5 in the IFT (including the SAN primordium) and atrium was significantly decreased at E8.75 and E9.5, respectively (E8.75, control: 96.46 ± 1.31%, mutant: 77.60 ± 4.12%, *n* = 4–5, *p* = 0.0057; E9.5: control: 89.10 ± 2.13%, mutant: 55.17 ± 6.50%, *n* = 4, *p* = 0.0025) (figure 4*e*–*i*).

*Cdk6*, a cyclin-dependent kinase gene promoting G1-S progress, is transactivated by Tbx5 in the pSHF during atrium development [11]. In the *Smo* mKO mutants, the expression of Cdk6 was downregulated (E8.75, control: 86.90 ± 2.14%, mutant: 68.52 ± 4.15%, *n* = 3, *p* = 0.0171; E9.5: control: 83.50 ± 0.07%, mutant: 59.09 ± 0.65%, *n* = 3, *p* < 0.0001) (figure 4*j*–*n*).

Hcn4 is required for the generation of pacemaker potentials in SAN cells [29]. Compared with that in the controls, the expression of Hcn4 in the IFT (including the SAN primordium) and atrium of the *Smo* mKO mutants markedly decreased at E8.75 and E9.5, respectively (E8.75, control: 82.86 ± 2.44%, mutant: 50.93 ± 4.07%, *n* = 3, *p* = 0.0025; E9.5: control: 89.77 ± 2.47%, mutant: 63.89 ± 1.10%, *n* = 3–4, *p* = 0.0004) (figure 4*o*–s).

To investigate the role of Hh signalling in SAN development, we dissected out the IFT (figure 4*t*) and examined the expression of the transcriptional factors critical for the SAN gene programme. Lineage tracing has revealed that the SAN develops from a subpopulation of Tbx3^+^ cells in the IFT [30]. Tbx3 is required for induction of the SAN gene programme [31]. The qRT-PCR results demonstrated that the expression level of *Tbx3* was significantly reduced in the IFT of the *Smo* mutant hearts (figure 4*u*). Shox2 expression was restricted to the sinus venosus, including the SAN and the venous valves of the developing heart. *Shox2* null mutants exhibit bradycardia and hypoplastic SAN [32]. In the *Smo* mKO mutants, *Shox2* expression was decreased (figure 4*v*). Tbx18 appears not to regulate the SAN gene programme but is required for SAN morphogenesis and deployment of the progenitors [33]. The qRT-PCR results indicated that the expression of *Tbx18* in the IFT of the controls was comparable to that in the mutants (figure 4*w*).

Isl1 acts upstream of the SAN signalling cascade to regulate pacemaker progenitor differentiation [34]. *Isl1* was detected in the IFT (SAN primordium domain, on the right side) and dorsal mesoderm in the controls at E9.5 (figure 4*x*), whereas its expression was decreased in the *Smo* mKO mutants at this stage (figure 4*x*). *Meis1* is associated with the PR interval [35], and its expression in the IFT was decreased in the mutants (electronic supplementary material, figure S6). *Myl7* is required for cardiomyocyte contraction, and its expression was reduced in the mutant IFT and atrium (electronic supplementary material, figure S6). Moreover, the inactivation of *Smo* in the mesoderm decreased the expression of *Arid3b* in the IFT (electronic supplementary material, figure S6).

Given that the genes critical for SAN development were downregulated in the mesodermal *Smo* knockout heart, we assessed the heart rate of E9.5 mouse embryos and found that the heartbeats were reduced in the newly dissected *Smo* mKO mutant embryos. We dissected the whole heart and studied the cardiac contractions in detail under a microscope. In the E9.5 control heart, the putative SAN beat rapidly (figure 4*y*; electronic supplementary material, Video S1, the dotted circles indicate the putative SAN), and the AV canal myocardium was also beating. In the E9.5 *Smo* mKO mutant heart, contraction of the putative SAN and AV canal myocardium was slower (figure 4*y*; electronic supplementary material, video S2, the dotted circle indicated the putative SAN). Statistical analyses showed that the heart rates of the control and *Smo* mKO mutants were 158 ± 14 b.p.m. and 49 ± 45 b.p.m. (*n* = 5, *p* = 0.0020), respectively (figure 4*z*). The significant difference in the cardiac rates demonstrated that cardiac conduction was impaired in the *Smo* mKO mutants.

Taken together, the results indicate that Smo controls the commitment of pSHF progenitors to the SAN cell lineage.

### 3.5. Smo maintains Bmp2 expression to induce EndMT during AV cushion formation

Bmp signalling is required for EndMT during AV cushion formation [17,36,37]. *Bmp2* is expressed in the AV myocardium from E8.5 to E10.5. The deletion of *Bmp2* with *Nkx2.5^Cre/+^* or Bmp type I receptor *Alk2* with *Tie2^Cre/+^* leads to a failed EMT [17,37].

**Figure 5.**
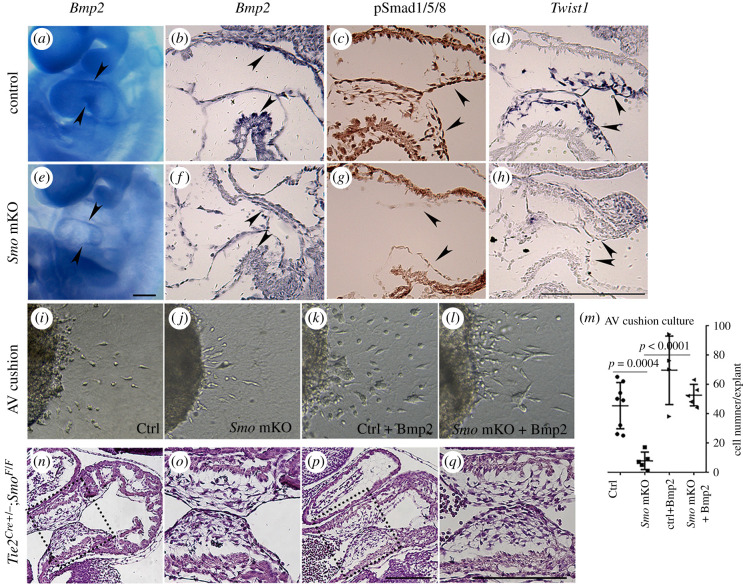
Hh signalling induces the EndMT via Bmp2 during AV cushion formation. (*a*–*h*) Reduced expression of *Bmp2* and related downstream molecules in the developing *Smo* mKO mutant hearts. Bmp2 mRNA expression in the myocardium (Ctrl: *a*,*b*; Mut: *e*,*f*; arrowheads), pSmad1/5/8 expression in the endocardium (Ctrl: *c*; Mut: *g*; arrowheads), and Twist1 mRNA expression in the endocardium and derived mesenchymal cells (Ctrl: *d*; Mut: *h*; arrowheads indicate the endocardium). (*i*–*l*) EndMT rescue assay with Bmp2 (Ctrl: *i*; *Smo* mKO mutant: *j*; Ctrl treated with Bmp2: *k* and *Smo* mKO mutant treated with Bmp2: *l*). (*m*) Quantification of the numbers of invaded mesenchymal cells in explant cultures showing the rescued EndMT in the AV cushion of the *Smo* mKO mutants treated with Bmp2. (*n*–*q*) AV cushion formation in the control is indistinguishable from that in the *Tie2^Cre/+^*; *Smo^F/F^* mutant. Scale bars: 200 µm.

We examined *Bmp2* expression in the developing hearts by ISH and found that the expression of *Bmp2* was reduced in the myocardium of the AV canal of the *Smo* mKO mutants at E9.0 (figure 5*a* and *b*, *e* and *f*). We further checked the level of phosphorylated-Smad1/5/8 (pSmad1/5/8) at E9.5 by immunostaining. In the control embryos, most endocardial cells and the overlying myocardium of the AV canal stained positive for pSmad1/5/8 (figure 5*c*), whereas in the *Smo* mKO mutant embryos, the staining was markedly reduced (figure 5*g*).

*Twist1*, encoding a basic helix–loop–helix transcription factor, is involved in the EndMT [37]. We thus examined the expression of *Twist1* in the AV cushion. In the controls, *Twist1* was expressed in the endocardium and derived mesenchyme of the AV canal (figure 5*d*). In the *Smo* mKO mutants, *Twist1* expression was diminished or markedly reduced at E9.5 (figure 5*h*).

To determine whether Bmp2 is sufficient for induction of the EndMT in the absence of Hh signalling, we performed a rescue assay in explant culture, a well-established model for studying the EndMT. In the control explants, a number of invasive mesenchymal cells were found in the collagen gel after 48 h in culture (figure 5*i*). By contrast, the mutant explants had fewer invasive mesenchymal cells (figure 5*j*). Furthermore, the addition of 100 ng ml^−1^ Bmp2 to the *Smo* mKO mutant explants significantly promoted invasive mesenchymal formation (figure 5*k*–*m*), which suggested that Hh signalling regulates the EndMT in the AV cushion by modulating *Bmp2* expression.

To test whether Smo expression in the endocardium is required for AV cushion formation, we ablated *Smo* specifically in endocardial/endothelial cell lineages using *Tie2^Cre/+^* mice. Interestingly, the mesenchymal cells in the AV cushion of the *Tie2^Cre/+^*;*Smo^F/F^* mutants formed with no notable defects by E9.75 (figure 5*n*–*q*, dashed boxes).

These results demonstrate that Smo signals in the myocardium of the AV cushion to regulate the expression of *Bmp2*, which induces the EndMT via lateral induction.

### 3.6. Analysis and identification of Hh signalling and its potential downstream targets in the pSHF

**Figure 6.**
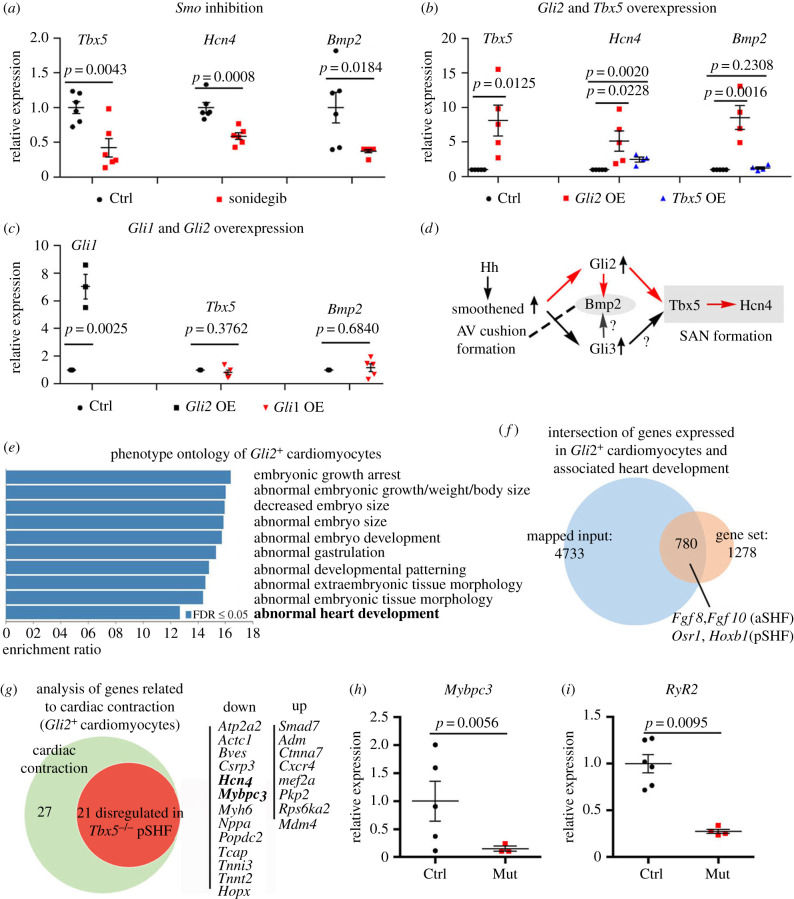
Regulatory pathway identification and analyses of potential downstream targets. (*a*) qRT-PCR results showing decreased expression of *Tbx5*, *Hcn4* and *Bmp2* in Smo-inhibited (sonidegib) cells. (*b*) The overexpression of Gli2 increased the expression of *Tbx5*, *Hcn4* and *Bmp2*, but Tbx5 enhanced the expression of *Hcn4* but not *Bmp2*. (*c*) The overexpression of Gli2 increased the expression of *Gli1*, and Gli1 overexpression did not change the expression of *Tbx5* and *Bmp2*. (*d*) Diagram showing the regulatory network revealed from the *in vitro* cell model. (*e*) Gene ontology functional cluster analysis of scRNAseq data from E8.5 *Gli2^+^* cardiomyocytes. (*f*) Venn diagram showing the gene expression profile in *Gli2^+^* cardiomyocytes. (*g*) Analysis of genes related to the cardiac contraction in *Gli2*^+^ cardiomyocytes. (*h*) Validation of downregulated expression of *Mybpc3* in the IFT of the *Smo* mKO mutant embryos. (*i*) Downregulated expression of *RyR2* in the IFT of the *Smo* mKO mutants.

To explore the gene regulatory network, we performed loss- and gain-of-function studies using the P19CL6 cell line, a well-established *in vitro* model for cardiomyocyte differentiation. In cells treated with sonidegib (a selective antagonist of Smo), the expression of *Tbx5*, *Hcn4* and *Bmp2* was decreased to 42.13 ± 32.34% (*n* = 6, *p* = 0.0043), 58.89 ± 11.84% (*n* = 6, *p* = 0.0008), 37.27 ± 6.12% (*n* = 5, *p* = 0.0184), respectively (figure 6*a*). Gli2 is the main effector of Hh signalling and is expressed in the cardiac mesoderm. In Gli2-overexpressing cells, the expression of *Tbx5*, *Hcn4* and *Bmp2* was increased to 811.20 ± 496.40% (*n* = 5, *p* = 0.0125), 513.80 ± 329.22% (*n* = 5, *p* = 0.0228), 854.75 ± 346.82% (*n* = 4, *p* = 0.0016), respectively. In the Tbx5-overexpressing cells, the expression of *Hcn4* was increased to 246.00 ± 69.53% (*n* = 4, *p* = 0.0020). However, Tbx5 did not affect *Bmp2* expression (figure 6*b*). These results suggested that Gli2 controls pacemaker progenitor cell differentiation by increasing *Hcn4* expression, at least in part via Tbx5 induction. Moreover, by examining the changed genes in the *Tbx5* mutant pSHF (RNAseq data) [21,38], we found that *Hcn4* expression in the pSHF was also reduced in the E9.5 *Tbx5* mutants. We further evaluated the regulation of Gli1 by Gli2. In Gli2-overexpressing cells, the expression level of *Gli1* was increased to 703.00 ± 154.00% (*n* = 3, *p* = 0.0025), whereas in Gli1-overexpressing cells, the expression levels of *Tbx5* and *Bmp2* were not altered (figure 6*c*).

Based on the results from our *in vivo* and *in vitro* studies, we propose the regulatory network model shown in figure 6*d*. In the presence of Hh morphogen, patched1 terminates its inhibition of Smo activity in the pSHF cardiac progenitors, leading to activation of the transcriptional factor Gli2. The Gli2 activator translocates to the nucleus to activate *Tbx5* and *Bmp2* expression. Tbx5 further activates the downstream gene *Hcn4*. The expression of *Bmp2* induced by Gli2 is Tbx5 independent.

Because *Gli2* is expressed in scattered cardiac progenitors, we analysed scRNAseq data of the E8.5 heart [39]. Among the total 109 cardiomyocytes, eight *Gli2^+^* positive cells were identified. The expression of *Tbx5*, *Hcn4* or *Bmp2* was detected in some *Gli2^+^* cardiac progenitors. GO functional cluster analysis revealed that approximately 780 genes expressed in *Gli2^+^* cardiac progenitors were involved in cardiac development (figure 6*e*,*f*). Forty-eight genes expressed in *Gli2^+^* cardiac progenitors were enriched in the cardiac contraction cluster. Of the 48 genes associated with cardiac contraction, 21 genes were down or upregulated in the pSHF of the *Tbx5^−/−^* mutants (figure 6*g*) [21]. Mutations in *Mybpc3* lead to abnormal cardiac muscle contraction and poor relaxation [40]. A scRNAseq analysis revealed that *Mybpc3* expression is downregulated in the *Tbx5^−/−^* mutant hearts (figure 6*g*). qRT-PCR analyses demonstrated that the expression of *Mybpc3* was reduced in the IFT of the *Smo* mKO mutant hearts (figure 6*h*). Type 2 ryanodine receptor (RyR2) controls calcium release, and *RyR2* mutations have been implicated in atrial fibrillation [41,42]. *RyR2* expression was markedly reduced in the IFT of the *Smo* mKO mutants (figure 6*h*).

Thus, we identified the Gli2–Tbx5–Hcn4 and Gli2–Bmp2 axes, which control SAN development and AV cushion formation, respectively, and we also analysed and validated the genes related to the cardiac contraction in *Gli2*^+^ cardiac progenitors.

## 4. Discussion

We have demonstrated that Hh signalling is required for the developmental potential of the cardiac progenitors and their differentiation towards pacemaker cells within the SAN and Bmp2^+^ cells within the AV canal myocardium.

### 4.1. Hh signalling controls the developmental potentials of cardiac progenitors in the pSHF

In this study, we found that the inactivation of *Smo* in the mesoderm reduces the de novo expression of *Tbx5* in the pSHF. *Wnt2*, a downstream target of Tbx5 [21], was also mildly decreased in the *Smo* mKO mutants. However, the loss of *Smo* did not affect the expression of *Hcn4* and *Isl1* at the approximately E7.5–E8.0 stage. These results demonstrated that Hh signalling is required for the developmental potentials but not the formation of the cardiac progenitors in the pSHF. It is reported that Tbx5 acts upstream and parallel to Hh signalling in the SHF [11]. The previous study [11] showed that Hh-dependent genes are downregulated at a later stage (E9.5) and that the defects are confined to the DMP in *Tbx5*^+/−^ mutants. Our data along with that obtained in the previous study suggests that the regulation of the Hh pathway by Tbx5 might constitute a feedback pathway.

### 4.2. Hh signalling controls SAN development and function by regulating genes critical for SAN progenitor commitment

A differential expression analysis of RNAseq data revealed that *Hcn4*, *Tbx3*, *Shox2*, *Isl1* and *Tbx18* are enriched in the SAN [43,44]. The SAN develops within the IFT domain and functions as a pacemaker. The electric impulses generated in pacemaker cells spread across the atrial myocardium to initiate contraction of the atria [33,45]. The activation pattern of the cardiac conduction has been established by E9.5 before the components of the cardiac conduction system are morphological recognized [33].

Tbx5 and Hcn4 are required for the specification and maturation of pacemaker progenitor cells, respectively [34]. Tbx3, Shox2 and Isl1 are also needed for SAN formation and conduction [33,34]. Tbx18 does not modulate the SAN gene programme but is needed for the formation of SAN progenitor cells [34]. In this study, we demonstrated that Hh signalling controls the expression of *Hcn4*, *Tbx3*, *Shox2* and *Isl1*. Furthermore, functional assay revealed that the heart rates are significantly decreased in *Smo* mutant heart. By contrast, the expression of *Tbx18* was not affected in the IFT of *Smo* mKO mutant heart. These results suggest that Hh signalling controls pacemaker progenitor cell commitment.

In addition, we measured cell proliferation at different embryonic stages. The proliferation of cardiac progenitor cells displayed a decreasing trend in the *Smo* mKO mutant hearts at E8.25–E8.5 (phospho-histone H3; controls: 3.11 ± 0.41%, mutants: 2.62 ± 0.38%, *n* = 3, *p* = 0.2057). By E9.5, a marked reduction of proliferation was found in the mutant hearts (EdU incorporation; control: 24.74 ± 0.83%, mutant: 15.34 ± 3.76%, *n* = 3–4, *p* = 0.0088) (electronic supplementary material, figure S7). It has been reported that *Tbx5* controls the expression of cell-cycle progression genes [11]. We thus reason that Hh signalling might also control cardiac proliferation through Tbx5.

### 4.3. Hh signalling is required for appropriate AV cushion formation by regulating *Bmp2* expression

Multiple signalling pathways are involved in the EndMT of the endocardium, and these pathways include Bmp/Tgfβ, Notch, Vegf and calcineurin/NFAT [46]. In this study, we demonstrated that Hh signalling controls myocardial *Bmp2* expression, which is required for activation of the Bmp pathway and initiation of the EndMT. Moreover, the administration of Bmp2 to the *Smo* mKO mutant AV cushion rescued the transition defect, which suggested that Bmp2 is both necessary and sufficient for induction of the EndMT by Hh signalling.

It has been reported that the specification of the AV cushion and the initiation of the EndMT proceed normally in *Shh^−/−^* hearts [10]. Shh is expressed in the notochord and node, and Ihh is expressed in the definitive endoderm and node [47]. *Shh* and *Ihh* compound mutants arrest shortly after gastrulation and phenocopy *Smo* mutants. *Shh* and *Ihh* compound mutants or *Smo* mutants exhibit a more severe phenotype than *Shh^−/−^* mutants [8]. We speculate that *Shh* and *Ihh* are both required for Smo-mediated EndMT during heart development.

### 4.4. Analyses of the Hh signalling pathway in the pSHF

We identified the Gli2Tbx5–Hcn4 axis which is essential for pacemaker progenitor cell differentiation and cardiac conduction. Although the expression of *Tbx5* in the pSHF was reduced in the *Smo* mKO mutants, its expression in the anterior heart field was less affected. These results suggest that the regulation of *Tbx5* by Hh signalling is context-dependent. We noted that the expression levels of *Hcn4* in the heart field were comparable between the control and *Smo* mKO mutant embryos at E8.0. As the embryo develops, the expression of *Hcn4* was decreased in the IFT and common atrium of the mutant hearts, which suggested *Tbx5* is required for the maintenance of *Hcn4* expression.

The cardiac progenitor cells in the pSHF contribute to the AV canal myocardium, atrium and IFT. The specific expression of Bmp2 in the AV canal myocardium induces the EndMT by lateral induction. We demonstrated that the overexpression of Gli2 enhanced *Bmp2* expression. The results indicate that Gli2 is required in cardiac progenitors for Bmp2^+^ cell lineage determination.

At E8.5 and E9.5. *Gli1-lacZ*, *Gli2-lacZ* and *Gli3* mRNA are dominantly expressed in the lateral plate mesoderm (pSHF) and contribute to atrium/IFT development [48]. In *Gli2^−/−^*;*Gli3^−/−^* double knockout embryos, the expression of *Tbx5* is reduced in the lateral plate mesoderm [48], which is consistent with our findings in *Smo* mKO mutants. These results indicate that *Gli2* and *Gli3* redundantly regulate the expression of *Tbx5* (figure 6*d*). In future study it would be interesting to explore whether Bmp2 expression is downregulated in the AV canal myocardium of the *Gli2* and *Gli3* compound mutant embryos. Gli1 and Gli2 exhibit similar expression patterns during cardiogenesis. *Gli1^zfd/zfd^* and *Gli1^lz/lz^* are viable with no obvious defects [49,50]. Both Gli1 and Gli2 were downregulated in the developing heart of the *Smo* mKO mutants (figure 4*a*–*d*), and the overexpression of Gli2 in P19CL6 cells increased the expression of Gli1 (figure 6*c*), which is consistent with the *in vivo* results [25]. Unlike Gli2, the overexpression of Gli1 did not alter the expression of *Tbx5* and *Bmp2* in the cell model (figure 6*c*). Thus, the *in vivo* and *in vitro* results indicate that Gli1 might not be essential for the formation of the SAN and AV cushion. In this study, we also found that Hh signalling controls other core transcriptional factors required for SAN node development. Single-cell ChIP-seq would be a powerful tool for dissecting the regulatory mechanism in the future.

We analysed scRNAseq data from the E8.5 mouse embryonic heart [39]. *Isl1*, *Tbx5*, *Hcn4*, *Mef2c*, *Fgf8*, *Wnt2*, *Osr1* and *Hoxb1* were expressed in *Gli2^+^* cells, which suggested that Hh responding cells contribute to both the aSHF and pSHF. Moreover, we predicted the potential downstream genes in the pSHF regulated by Gli2. Among these target candidates, *Hcn4* was validated by a gain-of-function assay. The qRT-PCR results demonstrated that the expression of *Mybpc3*, a potential downstream target gene, was reduced in the IFT of the *Smo* mkO heart. *Gli2^+^*, *Tbx5^+^*, *Shox2^+^* triple-positive cells were identified in E8.5 cardiomyocytes. We found that the expression of *Shox2* was reduced in *Tbx5^−/−^* and *Smo* mKO pSHF cardiomyocytes. The data suggest that Gli2 might regulate the expression of *Shox2* via Tbx5.

In summary, our data demonstrate that Hh signalling in the pSHF controls the activity of Gli2 to regulate the development of the SAN and the formation of the AV cushion.

## Data accessibility

Original data and materials are stored in our laboratory. Original data and materials will be available upon required.

The data are provided in the electronic supplementary material [51].
